# Case of Perforation of the Stomach

**Published:** 1829-10-01

**Authors:** 


PERFORATION OF THE INTESTINE.

The patient was an eminent banker, who did not abstain from his office till a day or two before his death. He was upwards of 70 years of age. The following very curious appearances presented themselves on examination after death.

On opening the cavity of the abdomen, it was discovered that the contents of the bowels had passed into it. On examining the intestines from the pylorus to the rectum, it was evident that recent inflammation had existed at various parts, but in general they were in a healthy state, until we arrived at the caput cæcum coli, which was thickened, and the whole gut from that portion was as enclosed in a case of organised coagulable lymph, with innumerable pouches, which appeared as if the colon in its natural state had escaped through the surrounding mass. On returning to examine the stomach, there was discovered near the pylorus a circular opening of about half an inch diameter externally, but on cutting into it, it was found that the inner and muscular coats of the stomach were destroyed to the extent of at least an inch—there was considerable thickness and hardness around the margin of what appeared to have been an ulcer, and through this opening the contents had escaped. There was a considerable quantity of fluid in the cavity of the abdomen—about four pints in the thorax—but not more than four ounces in the pericardium. The heart was enlarged and its substance thickened. The ascending aorta was dilated, and there was a disposition to ossification.

---

XXXI.

